# Sex-Specific Brain Responses to Imaginary Dance but Not Physical Dance: An Electroencephalography Study of Functional Connectivity and Electrical Brain Activity

**DOI:** 10.3389/fnbeh.2021.731881

**Published:** 2021-12-15

**Authors:** Johanna Wind, Fabian Horst, Nikolas Rizzi, Alexander John, Tamara Kurti, Wolfgang I. Schöllhorn

**Affiliations:** Department of Training and Movement Science, Institute of Sport Science, Johannes Gutenberg-University Mainz, Mainz, Germany

**Keywords:** EEG, sex differences, dance, physical activity, coherence, power spectrum, connectivity

## Abstract

To date, most neurophysiological dance research has been conducted exclusively with female participants in observational studies (i.e., participants observe or imagine a dance choreography). In this regard, the sex-specific acute neurophysiological effect of physically executed dance can be considered a widely unexplored field of research. This study examines the acute impact of a modern jazz dance choreography on brain activity and functional connectivity using electroencephalography (EEG). In a within-subject design, 11 female and 11 male participants were examined under four test conditions: physically dancing the choreography with and without music and imagining the choreography with and without music. Prior to the EEG measurements, the participants acquired the choreography over 3 weeks with one session per week. Subsequently, the participants conducted all four test conditions in a randomized order on a single day, with the EEG measurements taken before and after each condition. Differences between the male and female participants were established in brain activity and functional connectivity analyses under the condition of *imagined dance without music*. No statistical differences between sexes were found in the other three conditions (*physically executed dance with* and *without music* as well as *imagined dance with music*). Physically dancing and music seem to have sex-independent effects on the human brain. However, thinking of dance without music seems to be rather sex-specific. The results point to a promising approach to decipher sex-specific differences in the use of dance or music. This approach could further be used to achieve a more group-specific or even more individualized and situationally adapted use of dance interventions, e.g., in the context of sports, physical education, or therapy. The extent to which the identified differences are due to culturally specific attitudes in the sex-specific contact with dance and music needs to be clarified in future research.

## Introduction

Physical activity, particularly dance, is an increasingly popular field of research that shows, among other things, positive effects regarding wellbeing and health. Dance interventions led to improvements in association with dementia (Barnes et al., 2015), breast cancer (Sandel et al., 2005), autism (Koch et al., 2015), and Parkinson’s disease (Hackney and Earhart, 2009; Duncan and Earhart, 2012; Houston and McGill, 2013). Although these studies indicate positive effects, most research on dance has been conducted with fewer male than female participants (Brown et al., 2006; Cross et al., 2006, 2009; Orgs et al., 2008; Fink et al., 2009; Pilgramm et al., 2010; Jola et al., 2012; Cruz-Garza et al., 2014; Ermutlu et al., 2015; Müller et al., 2017; Poikonen et al., 2018b). Moreover, previous research neglected sex/gender differences in neurophysiological dance research, despite the widely accepted structural and functional brain differences between men and women. A large number of studies provided evidence that men have a larger brain volume, more white matter (Filipek et al., 1994; Cosgrove et al., 2007), a larger amygdala, and a larger hypothalamus (Kret and De Gelder, 2012) compared to women. Women have more gray matter volume (Gur et al., 1999; Cosgrove et al., 2007; Kret and De Gelder, 2012), a larger caudate, a larger hippocampus (Kret and De Gelder, 2012), and a greater corpus callosum. The greater corpus callosum in women indicates improved bi-lateralization with possible functional consequences (Rescher et al., 1999). Furthermore, women are associated with higher brain efficiency in the left brain regions (Gong et al., 2009), whereas men have increased right lateralization and higher right brain activation levels (Kret and De Gelder, 2012). Typically, the left hemisphere is associated with analytical processes, speech production, and comprehension. Visuospatial, analogical, intuitive, emotional, and subjective processes are more correlated to the right hemisphere (Rescher et al., 1999). Whether these structural brain differences correlate to differences in behavior between men and women has not been confirmed to date. It may be possible that brain structural differences compensate for genetic or hormonal differences and make the sexes more similar than different (Eliot, 2011; Kret and De Gelder, 2012; Oliver and Risner, 2017).

In addition to brain activity, there are disparities in functional connectivity between men and women. Widespread findings state that women have more inter-hemispheric connectivity in rest (Duffy et al., 1996; Rescher and Rappelsberger, 1996; Ingalhalikar et al., 2014; Jäncke, 2018), generate more positive correlations in the left hemisphere compared with the right hemisphere (Azari et al., 1992), and have greater functional connectivity in the left amygdala (subgenual cortex and hypothalamus) (Kilpatrick et al., 2006). In comparison, men display more connectivity in the right amygdala (sensorimotor cortex, striatum, and pulvinar) (Kilpatrick et al., 2006).

Only one study quantitatively examined sex/gender differences between males and females in the context of dance and its neurophysiology (Calvo-Merino et al., 2006). The study compared male and female expert dancers during an action observation of ballet moves and revealed an increased brain activity at the premotor, parietal, and cerebellar areas when watching the motor repertoire of their sex.

In addition to the lack of research regarding sex/gender differences, neurophysiological dance research has primarily focused on dance observation or the imagination of a dance (Cross et al., 2006; Orgs et al., 2008; Fink et al., 2009; Pilgramm et al., 2010; Jola et al., 2012; Bachrach et al., 2016; Poikonen et al., 2018a,b). So far, only two studies have investigated the acute effects of a physically executed dance on the brain using electroencephalography (EEG) (Cruz-Garza et al., 2014; Wind et al., 2020).

Considering the research deficits, the present study aims to investigate the neurophysiological effects of imagination and physical execution of a dance choreography (each with and without music) in men and women. The guiding research questions are:

(1) Does the electrical brain activity and functional connectivity differ between males and females after a physically executed and imagined modern jazz dance, with and without music?
(2) Do the test conditions, physically executing a modern jazz dance and imagining a modern jazz dance with or without music, differ in their influence on the electrical brain activity and functional connectivity?

With respect to our first research question, we assumed that the effects of the test conditions on brain activity and functional connectivity differ between men and women. This assumption is based on studies examining sex/gender differences in gross-motor activities, such as cycling. After cycling, female participants showed lower brainpower in the alpha frequency band at the frontal and central cortices, and lower temporal brainpower in the beta frequency band than male participants (Limb et al., 2014). Furthermore, during an incremental cycling test, the power in the alpha frequency band first increased in men continuing up to 80% of the test duration in the dorsolateral prefrontal cortex before decreasing. For women in comparison, the power in the alpha frequency band first decreased at 40% of the test before further significantly increasing (Dyrstad et al., 2019).

With respect to the second research question, we assumed differences in brain activity and functional connectivity between the physical execution and the imagination of the dance choreography. This assumption is predicated on the study by Wind et al. (2020) that revealed increased brainpower in the alpha and beta frequency band in frontal electrodes and an increase in the alpha frequency band in central electrodes after a physically executed dance choreography, while the imagination of the same choreography showed contrary effects. We further suspect differences between the test conditions with and without music. Since increased brainpower in the alpha frequency band in the temporal lobe was reported after physically performing the same dance choreography with and without music, the increase was significantly more pronounced after dancing without music (Wind et al., 2020). Further neurophysiological differences were reported in walking with and without music. Walking while listening to music increased brainpower in the beta frequency band in the frontal and frontal-central brain areas compared with walking without music (Bigliassi et al., 2018).

With the present study, we expanded the research on the neurophysiological effects of dance. It brings more insights into the neurophysiological sex/gender differences^1^ of dance since the given structural and functional sex/gender differences in the brain could be deepened by external influences, such as dancing, possibly leading to distinct effects in the brain of different sexes. To know how dance (styles) influences the brain of different sexes in a certain way allows more specific applications for individual needs. Neglecting sex/gender differences in e.g. needs-based therapies or training sessions could lead to undesirable consequences.

Next to the study aim of investigating sex/gender differences in the context of dance, it provides a valuable contribution on the effects of physically executed dance compared with imagined dance, since so far, previous research has in most cases focused on dance observation and imagination. Also, in this context, the knowledge of the effects of a body-performed dance could make therapies and training sessions more needs-oriented. As music is a core element in dance, its influence is examined as factor in isolation and combination with movement. Thus, this study intertwined two main aims, the possible different influences of dance on the brains of different sexes as well as the different impacts of a physically executed dance compared with an imagined dance on brain activity and functional connectivity.

More in-depth knowledge about these influences provides possibilities in the application of sports, dance therapy, and learning processes. Applications would be more precise, targeted, and could be tailored exclusively to individuals.

## Materials and Methods

### Participants

Eleven female and 11 male participants (mean age of women = 24.3 years, SD = 2.45; mean age of men = 26.2 years, SD = 3.74) volunteered for this study. None of the participants did dance in a dance association. All the participants were healthy, free from neurological disease, and right-handed. The participants were informed about the experimental procedure and provided with written informed consent forms prior to their participation. The design and conduction of the study were in accordance with the general principles outlined in the Declaration of Helsinki. The Local Ethics Committee of Johannes Gutenberg-University Mainz (Germany) approved the study.

### Experimental Procedure

**FIGURE 1:**
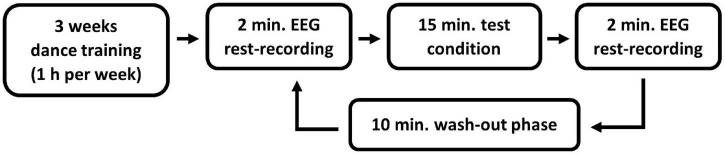
Overview of the experimental procedure.

Before the data acquisition, all the participants attended a 3-week sex-separated modern jazz dance training, consisting of a 1 h session each week in the sports facility of the Johannes Gutenberg-University Mainz. At the training sessions, all participants learned the same dance sequence with music. We selected a shortened version of the song “Black Out Days” by Phantogram. This song was chosen since the beat is audible and at a speed (92 bpm) where beginners can easily move. The choice of the dance style, modern jazz dance, was based on the physical education of the university, where it is one of the important contents of the education. A within-subject design with multiple pre-post-tests was chosen. Three to five days after the last training session, the EEG measurements of the individual test conditions were performed. All test conditions were performed by each participant within one day (refer to Figure 1 for an overview of the entire experimental procedure). The training sessions as well as the EEG measurements were conducted by a female investigator.

All tests and EEG measurements were conducted in a dimly lit room. Each test condition was preceded and followed by a 4 min resting condition (2 min eyes open and 2 min eyes closed), in which the brain activity was measured using EEG. Only the eyes-open resting condition was taken into account in the analysis to avoid the increase of alpha waves due to closed eyes (Barry and Blasio, 2017). A wash-out phase of 10 min took place between the individual test conditions (Bailey et al., 2008). During the resting and wash-out phases, the participants were asked to sit calmly on a chair facing a white wall.

Each participant accomplished every test condition for the modern jazz dance choreography in a within-subject design. Four test conditions had to be passed in a randomized order with opened eyes: (1) dance the modern jazz dance physically with music, (2) execute the same dance movements without music, (3) imagine the dance with music, and (4) imagine the dance without music.

### Data Acquisition

The EEG measurements were recorded with the Micromed SD LTM 32 BS amplifier (Venice, Italy) and the System Evolution Plus Software (Venice, Italy). Nineteen electrodes (Fp1, Fp2, F3, F7, Fz, F4, F8, C3, Cz, C4, T3, T4, P3, T5, Pz, P4, T6, O1, and O2) were applied according to the international 10–20-system with the reference electrode attached to the nose. The electrode impedances were kept below 10 kΩ. The EEG signals were digitized at a sampling rate of 1,024 Hz. The electrooculography (EOG) was affixed at the lateral orbital and the medial upper rim of the right eye.

### Data Pre-processing

For the EEG data pre-processing, a basic finite impulse response (FIR) bandpass filter was set to 2–41 Hz. An independent component analysis (ICA) was computed using the Matlab-based EEGLAB-toolbox 2019 (Delorme and Makeig, 2004). Interference-prone channels and recurrent movement artifacts, such as eye blinks and sweat artifacts, were deducted from the filtered signal based on the decision criteria that underline the ICLabel function from the EEGLAB-toolbox (Pion-Tonachini et al., 2017).

### Data Analysis

The measurements of the electrical brain activity were analyzed using the Matlab-based EEGLAB-toolbox mentioned above in the EEG data pre-processing [MathWorks, United States; Swartz Center of Computational Neuroscience, San Diego, CA, United States (Delorme and Makeig, 2004)]. Functional connectivity analyses were conducted using Matlab without further external toolboxes.

#### Electroencephalography Power Spectrum

The power spectrum was computed using the fast Fourier transformation with a Hanning window, a window size of 4,096 samples (4 s), and a window-overlap of 50%. The mean power spectra were calculated for the theta (3.5–7.5 Hz), alpha (7.5–12.5 Hz), beta (12.5–30 Hz), and gamma (30–40 Hz) frequency bands. These frequency ranges were progressed by Zschocke and Hansen (2012) as well as by Henz and Schöllhorn (2016) and Henz et al. (2018).

Since there are still discussions about the definition of frequency bands and their individuality and situatedness, we have also defined the frequency band limits individually on the basis of the individual alpha peak (IAP) (Kaiser, 2001; Klimesch, 2018).

We calculated the IAP at the O2 electrode for each participant, both before and after each test condition (1–4) of the rest-measurement (pre- and post-rest) with closed eyes (Bazanova, 2011). To consider differences of the IAP between the respective test conditions (1–4), measurement times (pre- and post-rest), as well as sexes, we calculated a three-factorial ANOVA. Neither the main nor the interaction effects showed statistically significant values. For the sake of simplicity, we only presented the characteristic values for the non-significant interaction effect, condition × time × sex [*F*(3,28) = 5.88, *p* = 0.628, $\eta_{p}^{2}=0.056$]. Based on these findings, we decided to conduct the analysis with the frequency band definitions according to Zschocke and Hansen (2012).

The power spectrum was analyzed for each of the four test conditions (physically executed dance with music, physically executed dance without music, imagined dance with music, and imagined dance without music), frequency band (theta, alpha, beta, and gamma), and brain lobe [frontal, central, temporal left, temporal right, and posterior (parietal and occipital)]. According to Lord and Opacka-Juffry (2016), the brain lobes were composed of the following electrodes: frontal *F* (Fp1, Fp2, F7, F3, Fz, F4, and F8), central (C) (C3, Cz, and C4), temporal left (*Tle*) (T3 and T5), temporal right (*Tri*) (T4 and T6), and posterior (*PO*) (P3, Pz, P4, O1, and O2).

#### Electroencephalography Functional Connectivity

The functional connectivity was analyzed using the magnitude-squared coherence (COH) (Shaw, 1984; Comani et al., 2013; Wang et al., 2015; di Fronso et al., 2018; Xiao et al., 2018). The COH describes the phase stability of two oscillating signals over a period of time (Guevara and Corsi-Cabrera, 1996). The main disadvantage of COH is the possible influence of volume conduction, which is understood as the reflection of a single source in multiple electrodes (Fein et al., 1988) and leads to zero-phase lagged signals. For this reason, we also analyzed functional connectivity by using the imaginary part of coherency (ICOH) (Nolte et al., 2004; Garcia Dominguez et al., 2013; Hohlefeld et al., 2013; Jamieson and Burgess, 2014; Lord and Opacka-Juffry, 2016). Since ICOH only considers the time-lagged signal parts, it is less sensitive to the assumed model of volume conduction (Nolte et al., 2004). However, ICOH seems to be a rigorous measure of connectivity, since not all zero-phase lagged signal parts might be caused by volume conduction. Similarly, if the signal source for volume conduction is not assumed to be centrally located, volume conduction can also lead to relative phase lags. In the current study, we analyzed both COH and ICOH but presented and interpreted solely the statistically significant results of COH analysis in the main text. The Supplementary Tables 1, 2 presents the associated pre- and post-rest measurements results of the ICOH analysis.

For a consistent value representation, the absolute value of ICOH was used, ranging from 0 to 1. In the case of COH, 0 means no interaction, and 1 means the complete or ideal interaction of two EEG electrodes. With the ICOH analysis, we can say with a higher probability that volume conduction had no influence, but it does not allow the unambiguous interpretation of a reduced or increased interaction. Coherency between frequency spectra *S*_*i*_(*f*) and *S*_*j*_(*f*) was calculated with the equation (1):


(1) $$C_{\mathrm{ij}}\,\left(f\right)=\frac{S_{\mathrm{ij}}\,\left(f\right)}{{\left[S_{\mathrm{ii}}\,\left(f\right)\,S_{\mathrm{jj}}\,\left(f\right)\right]}^{1/2}}$$

The cross-spectrum, *Sij(f)*, and the power-spectra, *Sii(f)* and *Sjj(f)*, were estimated using Welch’s method with the same window (size), overlap, frequency bands, and brain lobes as in the power spectrum analysis (see section “Electroencephalography Power Spectrum”).

### Statistical Analysis

The pre-processed data of the power spectrum and connectivity analysis were transferred to the SPSS-software (IBM, SPSS 23, Armonk, NY, United States) for a statistical evaluation.

The statistical differences between the female and male participants in the power spectrum and connectivity were calculated for each test condition, frequency band, and brain lobe using a Mann–Whitney *U* test since the data deviated significantly from a normal distribution (Shapiro–Wilk’s test). The sex/gender differences were calculated for pre-test data based on absolute power and connectivity values using a Mann–Whitney *U* test. The pre-tests revealed statistically significant differences between the male and female participants. Because of these differing initial activation levels, we restricted our between-sex/gender comparison in each condition on the difference values between pre- and post-rest.

Furthermore, the within-sex/gender differences between test conditions were calculated using a Friedman-test with Dunn–Bonferroni corrected *post hoc* tests. Since the pre-tests were statistically significantly different between test conditions, the analyses were calculated with different values too (pre-rest from post-rest subtraction in each test condition).

According to the Fisher statistics (Fisher, 1955), the statistical significance was set at a *p*-value ≤ 0.05. The effect size, mainly assigned to the Neyman–Pearson (1933) statistics, was converted to Cohen’s *r* (1992) with the following effect size thresholds: *r* = 0.1 (small effect), *r* = 0.3 (medium effect), *r* = 0.5 (large effect).

Since the power spectrum analysis compares five brain lobes and the connectivity calculation includes ten brain lobe pairs per frequency band and condition, the new significance level calculated with a false discovery rate (FDR)-correction is expected to be *p* ≤ 0.0021 in the power spectrum, and *p* ≤ 0.005 in connectivity. Due to the small *p*-values resulting from the high alpha correction, we only focused on sex/gender differences with *p* ≤ 0.05 with medium and high effect sizes (*r* ≥ 0.3). The decision against a strict adjustment according to Bonferroni can be supported by the criticism of this method (Perneger, 1998). In the case of the analyses between the test conditions, we focused on all ranges of the effect size.

## Results

### Power Spectrum

#### Between-Sex/Gender Differences in Test Conditions

**FIGURE 2:**
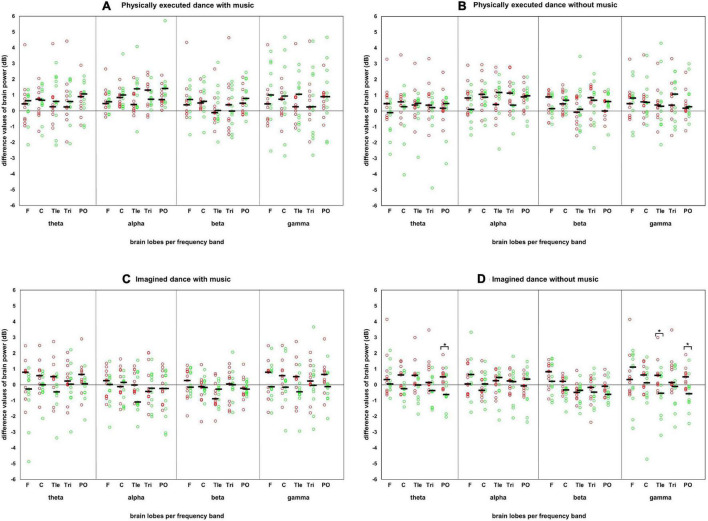
Difference values of each participant in the test conditions in the power spectrum. Difference values of each participant in each test condition, frequency band, and brain lobe in the power spectrum with the presentation of the median. Red circles: individual difference values of the female participants. Green circles: individual difference values of the male participants. Black horizontal bar: median of each sex/gender. Brain lobes: F, frontal lobe; C, central lobe; Tle, temporal left lobe; Tri, temporal right lobe; PO, posterior lobe. Four test conditions in panels: **(A)** physically executed dance with music, **(B)** physically executed dance without music, **(C)** imagined dance with music, and **(D)** imagined dance without music.

Table 1 shows the descriptive and inferential statistical values in the power spectrum analysis of the significant sex/gender differences. Differences were revealed in brainpower for the *imagined dance without music* condition in the theta frequency band at the PO and in the gamma frequency band in the temporal left *Tle* as well as in PO. In these test conditions with significant differences, the male participants showed a decreased and the female an increased brain power (see Figure 2).

**TABLE 1:** Significant between-sex/gender differences in test conditions in the power spectrum.

		Diff-rest 1 (dB)^b^	Increased/decreased 1^c^	Diff-rest 2 (dB)^b^	Increased/decreased 2^c^	*U* ^d^	*z* ^d^	*p* ^d^	*r* ^d^
		Women	Women	Men	Men				
**im^a^**									
Theta	PO	0.50	↑	−0.62	↓	30	−2.03	.045	0.43
Gamma	Tle	0.59	↑	−0.54	↓	30	−2.03	.047	0.43
	PO	0.50	↑	−0.57	↓	26	−2.27	.023	0.48

*Statistically significant differences between men and women in the power spectrum with different values. Left column, descriptive values; right column, statistical values.*

*^a^im, imagined dance without music.*

*^b^Diff-rest 1, difference value from pre- to post-rest-measurement in women; Diff-rest 2, difference value from pre- to post-rest-measurement in men.*

*^c^Arrow up, brainpower increased from pre- to post-rest measurement in women (increased/decreased 1) respectively in men (increased/decreased 2); arrow down, brainpower decreased from pre- to post-rest measurement in women (increased/decreased 1) respectively in men (increased/decreased 2).*

*^d^U-value and z-value of the Mann–Whitney U test, p-value, r-value of the effect size.*

#### Within-Sex/Gender Differences Between Test Conditions

The male participants (as shown in Table 2) displayed statistically significant differences in the alpha frequency band (PO) between the two test conditions: *physically executed with music* and *imagined dance with music*. The *physically executed dance with music*-condition showed increased brainpower in the alpha frequency band, and the *imagined dance with music* condition showed decreased brainpower in the alpha frequency band. Furthermore, differences could be observed between the *physically executed dance* and the *imagined dance without music* condition in the beta frequency band (C) in male participants. An increase of brainpower was observed in the *physically executed dance without music* and a decrease in the *imagined dance without music* condition. No statistically significant differences between the brainpower changes due to the test conditions were revealed for the female participants.

**TABLE 2:** Significant within-sex/gender differences between test conditions of the power spectrum in men.

Men		χ^2^ (df)^b^	Global *p*^b^	Diff-rest 1 (dB)^c^	Diff-rest 2 (dB)^c^	*Post hoc* comparisons		
						*p* ^b^	*z* ^b^	*r* ^b^
**da-m × im-m^a^**								
Alpha	PO	9.22 (3)	0.027	1.41	−0.24	0.030	2.81	0.85
**da × im^a^**								
Beta	C	9.87 (3)	0.02	0.68	−0.33	0.018	2.97	0.90

*Statistically significant differences between test conditions with the presentation of the Diff-rest values of the power spectrum. Left column, descriptive values; right column, statistical values.*

*^a^Statistical differences between test condition pairs, da-m, physically executed dance with music; da, physically executed dance without music; im-m, imagined dance with music; im, imagined dance without music.*

*^b^χ^2^ with degrees of freedom of Friedman-test, r-value effect size of the Friedman-test, z-value of the Friedman-test.*

*^c^Diff-rest 1, difference value from pre- to post-rest-measurement of the left test condition from test condition pair; Diff-rest 2, difference value from pre- to post-rest-measurement of the right test condition from test condition pair.*

### Connectivity Results

#### Between-Sex/Gender Differences in Test Conditions

Sex/gender differences were revealed in the COH in the *imagined dance without music* in the theta (C-Tle and Tle-PO), alpha (Tle-Tri and Tle-PO), and beta frequency band (F-Tri and Tle-Tri). Connectivity increased in all the frequency bands and lobes of the female participants, but decreased in males, except for the Tle–Tri interaction in the alpha frequency band where connectivity decreased in both sexes. The results separated for the COH and ICOH analyses are presented in Table 3 (COH) and Supplementary Table 1 (ICOH).

**TABLE 3:** Significant between-sex/gender differences in test conditions in coherence (COH).

im^a^		Diff-rest 1^b^	Increased/decreased 1^c^	Diff-rest 2^b^	Increased/decreased 2^c^	*U* ^d^	*z* ^d^	*p* ^d^	*r* ^d^
		Women	Women	Men	Men				
Theta	C-Tle	0.044	↑	−0.025	↓	29	−2.07	0.040	0.44
	Tle-PO	0.007	↑	−0.034	↓	27	−2.2	0.028	0.47
Alpha	Tle-Tri	−0.018	↓	−0.074	↓	26	−2.27	0.023	0.48
	Tle-PO	0.028	↑	−0.067	↓	26	−2.27	0.023	0.48
Beta	F-Tri	0.042	↑	−0.040	↓	28	−2.13	0.034	0.45
	Tle-Tri	0.005	↑	−0.040	↓	28	−2.13	0.034	0.45

*Statistically significant differences between men and women in COH. Left column, descriptive values; right column, statistical values.*

*^a^im, imagined dance without music.*

*^b^Diff-rest 1, difference value from pre- to post-rest-measurement in women; Diff-rest 2, difference value from pre- to post-rest-measurement in men.*

*^c^Arrow up, COH increased from pre- to post-rest measurement in women (increased/decreased 1) respectively in men (increased/decreased 2); arrow down, COH decreased from pre- to post-rest measurement in women (increased/decreased 1) respectively in men (increased/decreased 2).*

*^d^U-value and z-value of the Mann–Whitney U test, p-value, r-value of the effect size.*

#### Within-Sex/Gender Differences Between Test Conditions

Differences between test conditions could be identified for the male participants in the COH analysis between the *physically executed* and *imagined dance with music* in the alpha (C-Tri) and beta frequency band (C-Tri), with increased connectivity in the *physically executed dance with music* and a decrease in the *imagined dance with music.* Furthermore, differences could be identified between the *physically executed dance with music* and the *imagined dance without music* in the alpha (C-Tri and Tri-PO) and beta frequency band (C-Tri and Tri-PO). The results separated for the COH and ICOH analyses are presented in Table 4 (COH) and Supplementary Table 2 (ICOH). No differences between test conditions could be identified for the female participants.

**TABLE 4:** Significant within-sex/gender differences between test conditions of COH in men.

Men		χ^2^ (df)^b^	Global *p*^b^	Diff-rest 1	Diff-rest 2	*Post hoc* comparisons		
						*p* ^b^	*z* ^b^	*r* ^b^
**da-m × im-m^a^**								
Alpha	C-Tri	12.93 (3)	0.005	0.015	−0.036	0.010	3.14	0.95
Beta	C-Tri	11.18 (3)	0.011	0.035	−0.012	0.030	−2.81	0.85
**da-m × im^a^**								
Alpha	C-Tri	12.93 (3)	0.005	0.015	−0.041	0.018	−2.97	0.90
	Tri-PO	13.15 (3)	0.004	0.050	−0.050	0.010	3.14	0.95
Beta	C-Tri	11.18 (3)	0.011	0.035	−0.060	0.018	−2.97	0.90
	Tri-PO	08.02 (3)	0.046	0.048	−0.057	0.049	−2.64	0.80

*Statistically significant differences between test conditions with the presentation of the Diff-rest values of ICOH. Left column, descriptive values; right column, statistical values.*

*^a^Statistical differences between test condition pairs, da-m, physically executed dance with music; da, physically executed dance without music; im-m, imagined dance with music; im, imagined dance without music.*

*^b^χ^2^ with degrees of freedom of Friedman-test, r-value effect size of the Friedman-test, z-value of the Friedman-test.*

*^c^Diff-rest 1, difference value from pre- to post-rest-measurement of the left test condition from test condition pair; Diff-rest 2, difference value from pre- to post-rest-measurement of the right test condition from test condition pair.*

## Discussion

The present study examined the acute effects of a physically executed and imagined modern jazz dance choreography with or without music on electrical brain activity and functional connectivity. The focus was on the differences in the effects on male and female participants since sex/gender differences have been a neglected topic in neurophysiological dance studies. A further research interest referred to the comparison between an imagined and a physically performed dance since dance research concentrated predominantly on dance observation. The following discussion considers in two separate sections the statistically significant “Between-Sex/Gender Differences in the Test Conditions” and “Within-Sex/Gender Differences Between the Test Conditions.”

### Between-Sex/Gender Differences in Test Conditions

With respect to our first research question, we could confirm the assumption of sex/gender differences of the test conditions in brain activity and functional connectivity. The results indicate statistically significant sex/gender differences in neurophysiological effects following the test condition of *imagined dance without music*.

Overall, differences between the male and female participants occurred in all frequency bands. It is noticeable that while brainpower and functional connectivity increased in female participants, they decreased in male participants. Increased brainpower, especially following the *imagined dance without music*, can be interpreted to mean that imagining the dance without music initiated memory processes with a deep concentration in the female participants. This interpretation is based on the increased brainpower in the theta (PO) and gamma frequency band (Tle and PO) as well as on the increased connectivity in the theta frequency band (C-Tle and Tle-PO). These increases could be associated with increased memory processes {theta frequency (Klimesch, 1996); gamma frequency (Malik and Amin, 2017); and deep concentration [beta frequency (Abhang et al., 2016)]} on the imagination of the sequential organization [frontal lobe (Kolb, 2015)] of the rehearsed movements [central lobe (Thau et al., 2020)] in space [posterior and temporal lobe (Kolb, 2015; Brown and Chrastil, 2019)].

In addition, the increased connectivity in the alpha frequency band (Tle-PO) might be interpreted as a more relaxed brain state [alpha frequency (Zschocke and Hansen, 2012)] while imagining the dance in space [temporal lobe (Brown and Chrastil, 2019)] and still maintaining vigilance [posterior lobe (Husain and Nachev, 2007)]. Relaxed brain states might be beneficial in the context of an optimal learning state (Manuel et al., 2018) which could be of importance especially in educational contexts by, e.g., preparing cognitive lessons by dancing to achieve a supportive brain state for learning (Conyers and Wilson, 2015; Gutmann et al., 2015; Watson et al., 2017). However, the female participants could have also been drowsy or less concentrated after the *imagined dance without music* as the connectivity in the alpha frequency band [reduced concentration (Braboszcz and Delorme, 2011)] decreased (Tle-Tri), while the power as well as the connectivity in the theta frequency band [drowsiness (Kirschbaum, 2008)] increased (PO and Tle-PO). The repetitive imagination of the choreography in space [temporal and posterior lobe (Kolb, 2015; Brown and Chrastil, 2019)] might have led to drowsiness, and thus to a reduced concentration.

The increased brainpower in the gamma frequency band (Tle) might be related to emotional [temporal (Simons and Johnsrude, 2014)] memory processes [gamma frequency (Malik and Amin, 2017)], in the manner of, e.g., stress or excitement. The female participants could have been stressed [gamma frequency (Abhang et al., 2016)] due to the repetitive imagination of the choreography, the claim to fulfill the task in perfection, the unknown premises, or due to maintaining vigilance [parietal lobe (Husain and Nachev, 2007)]. These processes might be mirrored in increased brainpower in the temporal lobe, characterized by emotion processing (Simons and Johnsrude, 2014). The increased connectivity in the beta frequency band (Tle-Tri) could further underpin the stress-processing [beta frequency (Jena, 2015)]. Otherwise, the female participants could have also been excited [beta frequency (Lu et al., 2005)] since they possibly performed the dance to their satisfaction.

In comparison, the decreased brainpower in the theta (PO) and gamma frequency band (Tle and PO) in the male participants could be interpreted as the reduced use of memory processes [theta (Klimesch, 1996) and gamma frequency (Müller et al., 2000)] after the imagination of the dance without music. The decreased connectivity in the theta (C-Tle and Tle-PO), alpha (Tle-Tre and Tle-PO), and beta frequency band (F-Tri and Tle-Tri) could underpin these lower memory processes due to its association to a reduced concentration [theta (Klimesch, 1996), alpha and beta frequency (Braboszcz and Delorme, 2011)]. Moreover, the repetition could have also been more relaxing since the brainpower in the stress-connected gamma frequency band (Abhang et al., 2016) decreased and the required task resembles meditation (Hinterberger et al., 2014; DeLosAngeles et al., 2016). However, since connectivity decreased in the beta frequency band (Tle-Tri) in the *imagined dance without music* in the male participants, the presence of mental fatigue (Xu et al., 2018) or boredom (Katayama and Natsume, 2012) could be assumed, too.

Since statistically significant differences between the female and male participants could be observed in the *imagined dance without music*, it is recommended for future dance observation/imagination studies to consider the different neurophysiological reactions of the sexes. No statistically significant sex/gender differences could be observed in the *physically executed dance with* and *without music*. It seems like the neurophysiological reaction of the sexes on performing a dance were more similar than different, which should be considered, e.g., in the application of dance therapy. However, indicators for sex/gender differences were found and could confirm the first assumption partially.

### Within-Sex/Gender Differences Between Test Conditions

Statistically significant differences between the test conditions were identified in the brainpower of the male participants, both between the *physically executed* and *imagined dance with music*, as well as between the *physically executed dance* and the *imagined dance without music*. Additionally, differences were identified in terms of the functional connectivity of the male participants between the *physically executed* and *imagined dance with music* as well as between the *physically executed dance with music* and the *imagined dance without music*. These findings answer our second research question and could confirm the assumptions of differences between the test conditions.

The increased brainpower in the alpha frequency band (PO) and the connectivity in the alpha and beta frequency band (C-Tri) in the *physically executed dance with music* compared with the *imagined dance with music* could be interpreted as a relaxed brain state [alpha (Zschocke and Hansen, 2012)] and an increased focus on the execution of the movements [central lobe (Thau et al., 2020)] in space [temporal and posterior lobe: spatial navigation (Kolb, 2015)]. However, the increases in the connectivity in the beta frequency band (C-Tri) could also be assigned to stress or excitement (Lu et al., 2005; Jena, 2015). Thus, the female participants might have been stressed due to the repetitive movements, the aim to dance in perfection, or due to unknown premises. However, they also might have been excited, since they were looking forward to dancing the choreography they learned in the prior training sessions (Lu et al., 2005). The decreased brainpower and connectivity in the *imagined dance with music* could be traced back to reduced concentration (Braboszcz and Delorme, 2011; Schapkin et al., 2020) or boredom (Katayama and Natsume, 2012; Fakhr Tabatabaie et al., 2014) as they repetitively imagined the choreography.

Regarding the differences between the *physically executed dance* and the *imagined dance without music*, the increases in the brainpower in the *physically executed dance without music* could be interpreted as a higher cortical effort [beta frequency (Zschocke and Hansen, 2012)], stress (Jena, 2015) or excitement (Lu et al., 2005). The involvement of the central lobe might mirror processes of movement execution (Thau et al., 2020). In comparison, the decreases in the brainpower in the *imagined dance without music* might be denoted to a reduced concentration [beta frequency (Braboszcz and Delorme, 2011)] or boredom (Katayama and Natsume, 2012; Fakhr Tabatabaie et al., 2014) with the same interpretive approaches as between the *physically executed* and *imagined dance with music*.

The increased connectivity in the alpha and beta frequency band (C-Tri) in the *physically executed dance with music* compared with the *imagined dance without music*, could be interpreted as mentioned above (relaxation, stress, and excitement). Also, the decreased connectivity in the *imagined dance without music* might indicate reduced concentration (Braboszcz and Delorme, 2011) or boredom (Katayama and Natsume, 2012; Fakhr Tabatabaie et al., 2014). Further, the interaction between the Tri and PO in these two frequency bands and test conditions could support an increased, respectively, decreased processing of spatial navigation (Kolb, 2015). Moreover, the increased connectivity in the *physically executed dance with music* between these brain lobes might indicate the processing of auditory inputs like the music (temporal lobe), combined with processes of spatial navigation (PO) while dancing (Kolb, 2015).

### Limitations and Future Work

Our results indicate differences in the neurological effects of the imagination of a dance between men and women and between test conditions in all frequency bands. However, there are multiple interpretations possible for these differences found and more specific studies with additional measures on the level of relaxation, excitement, stress, or boredom need to examine these differences in more detail. Whether our findings are related to sex-specific anatomical (Filipek et al., 1994; Cosgrove et al., 2007; Kret and De Gelder, 2012) or neurophysiological characteristics (Duffy et al., 1996; Rescher et al., 1999; Gong et al., 2009), or sociocultural expectations (e.g., dance affinities and music preferences) requires further research in the future.

In this context, the sex differences could also be attributed to the influences of menstruation on women, which were not considered in this study. A wide variety of studies have shown that the menstrual cycle affects motor execution (Carmichael et al., 2021) and brain activity (Solis-Ortiz et al., 1994, 2004), as well as functional connectivity (refer for review McEwen and Milner, 2017). For example, the female hormone, estrogen, can increase the excitability of the brain in female cats (Alcaraz et al., 1969) and should therefore be investigated further in future studies. However, other studies suggest only little/no influence of the menstrual cycle on electrical brain activities, resulting in no significant differences between men and women due to the female menstrual cycle (Le et al., 2020). Therefore, future studies might clarify whether the hormonal cycle of the sexes leads to significant differences in the brain activity. It needs to be clarified if brain activity is more strongly influenced by menstrual fluctuations than by individual fluctuations (such as circadian rhythms, emotions, and fatigue), to allow conclusions on differences between the sexes. Furthermore, it has to be clarified to what extent these influences (fatigue, menstrual cycle, and emotions) can be detected in the IAP. Thereby, sex analysis regarding brain activity is considered to be only the beginning of the differentiation on the way to the individual. The collection of all these influences could enable a specialized analysis and thus support a very differentiated diagnosis for personalized interventions.

In addition, the methodological limitations are listed below. Since this study presents a coarse approach on sex/gender differences in the context of dance at the brain level, determinations had to be made on a specific dance style as well as music to set a beginning of this field of investigation. Correspondingly, the experimental procedure features 3 weeks of training, after which the participants were able to dance the choreography automatically and without the feedback of the instructor. The extent to which further influences, such as side effects like patterning and movement memory, were investigated too, must be clarified in detail in future studies. The influence of the trainer’s sex (here female) (Good et al., 1973; Kanedi and Sutyarso, 2014) could be seen as a factor worth being investigated in the future, especially related to teaching dance dependent on cultural differences. Moreover, the musical and movement preferences of the experimental conductor might have contrasted with the individual preferences of the participants.

With regards to the tending higher median in the physically executed dance compared with the imagined dance test conditions (as shown in Figure 2), it should be noted that with the current study design we cannot specify whether the neurophysiological effects are dance-specific or occurred due to the higher physical effort during the movement execution (John et al., 2020). Future research should therefore compare dancing with other types of exercise (e.g., running and pedaling) under comparable physical demands. Additionally, it should be critically noted that there was no objective verification of the thoughts of the participants during the *imagined dance without music*.

Moreover, the current study points to the need for research on the individual (see Figure 2), since several participants reacted individually to the different test conditions, deviating from the group median (refer to difference values in Figure 2). Thus, the analysis of the individual and, one step further, of the situation is recommended in future studies.

In addition, we need to consider that the functional connectivity results solely from the COH analysis were presented and interpreted in the main text. Although the main disadvantage of the COH analysis is the possible influence of volume conduction, its values range from 0 to 1 can be interpreted without ambiguity. For the reason of volume conduction, we further analyzed the ICOH to circumvent the zero-phase lagged signals and thus the volume conduction (Nolte et al., 2004). However, the results of the ICOH did not allow a clear interpretation of an increase or decrease in the interaction between the two signals. Accordingly, the statistically significant results from the COH analysis were presented with the pre- and post-rest measurement values of the ICOH analysis in Supplementary Tables 1, 2. The larger the difference from the pre- to post-rest measurement value of the ICOH, the less likely the occurrence of volume conduction should be and vice versa under the assumption that the signals have a common source with equal distance to the measuring electrodes. Taking into account the layer-specific anatomical connectivity of the gray matter, this assumption seems questionable and oversimplifies the actual problem (Jirsa and McIntosh, 2007; Wang et al., 2014; Gilbert and Baresi, 2016). Thus, these values could be used to verify whether the statistically significant results of the COH can rather be attributed to volume conduction or an actual brain interaction.

## Conclusion

This study represents an approach to the topic of sex/gender-specific differences in brain activity in the context of dance. The data analysis revealed an increased electrical brain activity and functional connectivity in the *imagined dance without music* in the male participants compared with the female. Differences in the neurophysiological effects between the male and female participants occurred in all frequency bands, indicating the differences in relaxation, concentration, stress, excitement, or boredom. Due to the pilot character of the study and the chosen statistics, no claim for generalization is made. Consequently, the study has limitations that are worth considering in follow-up studies. The aspects to be investigated in the future could include the frequency band adjustments, sociocultural expectations, low amplitude electromyography (EMG) contaminations, circadian fluctuations of the hormones, emotions, and fatigue. The extent to which the type of movement and the style of music play a role in individual memory formation must additionally be considered separately and investigated specifically, as must their interactions with the previously mentioned aspects. Future research will need to fathom the found in more detail, with more specific studies in terms of the different possible interpretations for the differences found and examine whether these observations are associated with, e.g., neurophysiological characteristics or sociocultural expectations. Research and therapeutic approaches should consider the sex/gender-specific differences of an imagined dance and similarities of a physically executed dance (with or without music) in the future; thus, applications can be more targeted, specific, and individualized to different patients. However, the differentiation between men and women can be considered as the first step from generalization to more specificity. The next logical step points towards person-specific analyses for even more differentiated, tailor-made applications in therapy and teaching.

## Data Availability Statement

The original contributions presented in the study are included in the article/Supplementary Material, further inquiries can be directed to the corresponding author.

## Ethics Statement

The studies involving human participants were reviewed and approved by Local Ethics Committee of Johannes Gutenberg-University Mainz (Germany). The patients/participants provided their written informed consent to participate in this study.

## Author Contributions

JW, FH, NR, AJ, TK, and WS collaborated in preparing the manuscript. JW and WS designed the experiment. JW and TK conducted the data acquisition. JW implemented data processing and statistical analysis and wrote the manuscript. FH and NR contributed the analysis tools. JW, AJ, NR, and WS interpreted the data. FH, AJ, NR, and WS critically revised the manuscript. All authors contributed to the article and approved the submitted version.

## Conflict of Interest

The authors declare that the research was conducted in the absence of any commercial or financial relationships that could be construed as a potential conflict of interest.

## Publisher’s Note

All claims expressed in this article are solely those of the authors and do not necessarily represent those of their affiliated organizations, or those of the publisher, the editors and the reviewers. Any product that may be evaluated in this article, or claim that may be made by its manufacturer, is not guaranteed or endorsed by the publisher.

## Footnotes

1 Throughout this article, the term “sex/gender differences” will refer to Jäncke (2018), despite these terms having different definitions. Sex refers to an individual’s reproductive system and secondary sexual characteristics. Gender means the social role of an individual based on sex or personal identification. While the structural and functional brain differences based on sex or gender cannot be investigated in this article, the combined term “sex/gender differences” is used in the following.
