# Supplementary material for: Sex-Specific Brain Responses to Imaginary Dance but Not Physical Dance: An Electroencephalography Study of Functional Connectivity and Electrical Brain Activity
Source: Front Behav Neurosci. 2021 Dec 15;15:731881. doi: 10.3389/fnbeh.2021.731881 (PMC8715740; doi:10.3389/fnbeh.2021.731881)
Supplement: Supplementary file 1 [file Table_1.pdf]

Supplementary Material

Table 1. Between-sex/gender differences in test conditions in ICOH.

		pre-rest [b] women	post-rest [b] women	in-/decreased[c] women	pre-rest [b] men	post-rest[b] men	in-/decreased[c] men
**im[a]**							
Theta	C-Tle	0.086	0.080	↓	0.058	0.079	↑
	Tle-PO	0.079	0.080	↑	0.056	0.070	↑
Alpha	Tle-Tri	0.088	0.087	↓	0.089	0.091	↑
	Tle-PO	0.090	0.106	↑	0.072	0.078	↑
Beta	F-Tri	0.077	0.074	↓	0.081	0.080	↓
	Tle-Tri	0.073	0.071	↓	0.085	0.078	↓

Note: Pre- and post-rest measurement results of ICOH belonging to the statistically significant differences between men and women in COH. Left column – statistically significant different electrode pairs of COH, middle column – pre- and post-rest measurement values of women, right column – pre- and post-rest measurement values of men.

[a]im: imagined dance without music

[b]pre- and post-rest: pre- and post-rest-measurement value in women and men.

[c]arrow up: ICOH increased from pre- to post-rest measurement in women resp. in men, arrow down: ICOH decreased from pre- to post-rest measurement in women resp. in men

Table 2. Within-sex/gender differences between test conditions in ICOH in men.

		pre-rest 1 [b]	post-rest 1 [b]	in-/decreased 1[c]	pre-rest 2 [b]	post-rest 2[b]	in-/decreased 2[c]
**da-m*im-m[a]**							
Alpha	C-Tri	0.103	0.122	↑	0.094	0.092	↓
Beta	C-Tri	0.082	0.091	↑	0.074	0.079	↑
**da-m*im[a]**							
Alpha	C-Tri	0.103	0.122	↑	0.092	0.103	↑
	Tri-PO	0.093	0.102	↑	0.086	0.080	↓
Beta	C-Tri	0.082	0.091	↑	0.075	0.081	↑
	Tri-PO	0.067	0.075	↑	0.068	0.071	↑

Note: Pre- and post-rest measurement results of ICOH belonging to the statistically significant differences between test conditions of the COH in men.

Left column – statistically significant different electrode pairs of COH in men between test conditions, middle column – pre- and post-rest measurement values of test condition 1, right column – pre- and post-rest measurement values of test condition 2.

[a]Test condition pairs, da-m: physically executed dance with music, im-m: imagined dance with music, im: imagined dance without music

[b]Pre- and post-rest 1: pre- and post-rest-measurement value of the left test condition from test condition pair, pre- and post-rest 2: pre- and post-rest-measurement value of the right test condition from test condition pair.

[c]arrow up: ICOH increased from pre- to post-rest measurement of the left resp. right test condition from test condition pair, arrow down: ICOH decreased from pre- to post-rest measurement of the left resp. right test condition from test condition pair.
